# Melatonin-Attenuated Oxidative Stress in High-Risk Pregnant Women Receiving Enoxaparin and Aspirin

**DOI:** 10.1155/2023/9523923

**Published:** 2023-05-25

**Authors:** Nahid Azarmehr, Roghayeh Porhemat, Narges Roustaei, Esmat Radmanesh, Zahra Moslemi, Razieh Vanda, Zahra Barmoudeh, Parvinsadat Eslamnik, Amir Hossein Doustimotlagh

**Affiliations:** ^1^Medicinal Plants Research Center, Yasuj University of Medical Sciences, Yasuj, Iran; ^2^Student Research Committee, Yasuj University of Medical Sciences, Yasuj, Iran; ^3^Department of Biostatistics and Epidemiology, School of Health, Yasuj University of Medical Sciences, Yasuj, Iran; ^4^Department of Physiology, Faculty of Medicine, Abadan University of Medical Sciences, Abadan, Iran; ^5^Clinical Research Development Unit, Imam Sajad Educational Hospital, Yasuj University of Medical Sciences, Yasuj, Iran; ^6^Department of Obstetrics and Gynecology, Imam Sajad Hospital, Yasuj University of Medical Sciences, Yasuj, Iran; ^7^Department of Clinical Biochemistry, Faculty of Medicine, Yasuj University of Medical Sciences, Yasuj, Iran

## Abstract

**Objective:**

In pregnancy, reducing inflammation and oxidative stress is important. Administration of melatonin during pregnancy can improve reproductive performance by improving the placental antioxidant system and inflammatory response. This investigation was carried out to evaluate the beneficial impact of melatonin on the oxidative stress state among high-risk pregnant women receiving enoxaparin and aspirin.

**Methods:**

In this double-blind, placebo-controlled trial, 40 pregnant women, aged 15–45 years at 6 weeks of pregnancy, were randomly selected and divided into intervention and control groups. The control group received prophylaxis enoxaparin and aspirin once daily between 6 and 16 weeks of pregnancy. The intervention group was taken enoxaparin and aspirin for 9 weeks and melatonin once daily from the sixth week of pregnancy to delivery time. Blood samples were taken to measure some oxidative stress biomarkers including total antioxidant capacity (TAC), malondialdehyde (MDA), total thiol (T-SH), protein carbonyl (PCO), and nitric oxide (NO). The level of high-sensitivity C-reactive protein (hs-CRP) was also determined.

**Results:**

TAC and T-SH levels increased significantly in the intervention group in comparison with the control group. Melatonin administration compared to the control group led to a significantly decreased level of NO and an insignificant hs-CRP level.

**Conclusion:**

Melatonin supplementation in high-risk pregnancy had favorable effects on TAC, T-SH, NO, and hs-CRP levels, improved antioxidant activity, and reduced inflammation. More studies are needed in different pregnancy conditions along with the measurement of different biomarkers.

## 1. Introduction

Pregnancy is a physiological state associated with increased oxidative stress resulting from increased metabolism and oxygen demand of placental and fetal tissue [1]. Furthermore, oxidative stress is implicated in hypoxia of compromised pregnancies such as preeclampsia (PE), gestational diabetes mellitus, and maternal undernutrition [2, 3]. Several studies have indicated that expanded maternal oxidative stress due to increased reactive oxygen species (ROS) production and impaired antioxidant defense mechanisms is related to fetal growth retardation. These detrimental impacts of maternal oxidative stress on birth results have been studied by evaluating the maternal oxidative stress status at different times throughout pregnancy [4]. At first, the placenta has a low-oxygen environment, and as it matures and its vessels develop, it changes to an oxygen-rich environment and its abundant mitochondrial mass increases the production of ROS. Nitric oxide (NO) is also produced locally by the placenta contributing to the progress of oxidative stress [5]. While antioxidant activity gradually becomes desirable over oxidation during normal pregnancy, it appears to be an inadequate increase in antioxidants to neutralize the elevated free radicals production [6]. Controlling inflammation and oxidative stress is a critical part of a healthy pregnancy. Nevertheless, when inflammation is dysregulated, it puts pregnancy at risk owing to some downstream physiological consequences, such as disorders of immune function, fetal development, endocrine activity, and vascular physiology [7]. Tumor necrosis factor-alpha (TNF-*α*) and hs-CRP are common markers indicating elevated systemic inflammation and are associated with disease in pregnancy [8]. Some investigations showed that the use of low molecular weight heparin (enoxaparin) and aspirin to improve pregnancy outcomes has not been so favorable [9, 10]. Therefore, the reduction of adverse complications resulting from inflammation and oxidative stress in pregnant women requires applying various strategies including antioxidant supplementation and using oxidative stress-lowering and anti-inflammatory compounds [11].

Melatonin (5-methoxy-*N*-acetyltryptamine) is an endogenous lipid-soluble hormone, predominantly produced in the pineal gland that is best recognized for its role in providing circadian and seasonal timing cues [12]. Additionally, it has been known as a potent antioxidant. The mechanism of its performance includes scavenging free radicals, inducing antioxidant enzymes, such as glutathione reductase and glutathione peroxidase, as well as inhibiting the prooxidative enzyme nitric oxide synthase (NOS). Melatonin supplementation for pregnancy has been investigated for a wide range of conditions [13]. Melatonin administration during pregnancy could ameliorate maternal–placental–fetal redox status and reproductive performance by improving the placental antioxidant system and inflammatory response [14]. Also, melatonin is important in high-risk pregnancy for its potent antioxidant and anti-inflammatory effects, genic receptor expression, and regulation of the circadian rhythm [15]. Considering the increase in oxidative stress and inflammation in women with high-risk pregnancy and considering the antioxidant and anti-inflammatory properties of melatonin and the fact that the effect of this supplement on high-risk pregnancy has not been investigated, this study aims to investigate the effect of melatonin on the level of oxidative stress and inflammatory markers in high-risk pregnant women receiving enoxaparin and aspirin.

## 2. Materials and Methods

This randomized, double-blind, placebo-controlled trial was conducted in *Imam Sajad Hospital, Yasuj, Iran,* from 2020 to 2022. Forty persons in the age range of 15–45 among high-risk pregnant women (based on the Venous thromboembolism risk score during pregnancy) [16] receiving prophylaxis enoxaparin and aspirin from the sixth to sixteenth week's gestation were recruited in this study. Exclusion criteria in this study are pregnant women with the following diseases: diabetes mellitus, high blood pressure, metabolic diseases, rheumatological diseases, bone diseases, hemolytic diseases, endocrine diseases, and malabsorption, as well as pregnant women with a history of using corticosteroids, ocular cicatricial pemphigoid (OCP), levothyroxine, progesterone, supplements (such as iron, calcium, and folic acid), smoking, other addictive substances, and alcohol consumption. Finally, a total of 40 pregnant women remained in the study. Participants were randomly divided into intervention and control groups (*n* = 20 patients per group). The study was carried out based on the guidelines set out in the Declaration of Helsinki. The Ethics Committee of the *Yasuj University of Medical Sciences* approved the study, and written informed consent was acquired from all subjects. The study is registered in the Iranian Registry of Clinical Trials (IRCTID: IRCT20210608051518N1, https://www.irct.ir/trial/60425).

### 2.1. Study Design

The control group received prophylaxis enoxaparin and aspirin once daily between 6 and 16 weeks of pregnancy. The intervention group took enoxaparin and aspirin between 6 and 16 weeks of pregnancy and melatonin once daily from the sixth week of pregnancy to delivery time. In this study, the assignment of subjects to the two intervention or control groups was unknown to the patients and the investigator, and the coding program was conducted by a third person who was unfamiliar with the study.

Venous blood samples (10 ml) were taken 48–72 hours before the start of administration (baseline) and after delivery, during the study period. The blood samples were centrifuged at 3500 rpm for 10 minutes, and the serum was aliquoted and stored at −20°C for evaluation of parameters related to oxidative stress and inflammation such as MDA, TAC, T-SH, NO metabolite, protein carbonyl, and hs-CRP. Serum hs-CRP concentration was measured using the ELISA kit. The nitrite level was determined as an index of NO production using the Griess method [17]. The plasma level of TAC was assayed by the ferric-reducing ability of plasma (FRAP) method suggested by Benzie and Strain [18]. The protein carbonyl test was performed by a spectrophotometric method [19]. The plasma MDA level was estimated using the TBA reaction assay [20]. The total thiol (T-SH) level was measured based on the reaction with DTNB [21].

### 2.2. Statistical Analyses

The data were analyzed using SPSS software using descriptive and inferential statistics. The normality of quantitative data was investigated using the Shapiro–Wilk test. An Independent *t*-test was used to compare the mean of normal variables between the two groups, and the paired *t*-test was utilized to compare the scores of variables before and after administration in each group. In cases of non-normality of data distribution, the significance of the difference in the average change after administration compared to the baseline state in each group was performed using the Wilcoxon test, and the Mann–Whitney was used to compare the two groups.

## 3. Results

### 3.1. Effect of Melatonin on Oxidative Stress and Inflammatory Parameters

As shown in Table 1, TAC and T-SH levels increased significantly in the intervention group in comparison with the control group. However, the administration of melatonin compared to the baseline value and the control group led to a considerable decrease in levels of serum NO (*p* = 0.004). We did not observe a significant effect of melatonin on plasma MDA levels. The PCO content decreased significantly in both the intervention and control groups compared with the baseline value. However, the reduction in the control group was more obvious compared to the melatonin supplementation group. The CRP level reduced dramatically in the intervention group at the end of the study (4.38 ± 0.87) in comparison with the basic feature (12.85 ± 9.67). Nevertheless, this alteration was not considerable compared to control subjects.

## 4. Discussion

Gestation is a physiological condition related to elevated metabolic demands and an increased need for tissue oxygen. This increased oxygen demand strengthens the rate of ROS generation, particularly in the second half of pregnancy. The last trimester of gestation is an important time of rising fat catabolism, insulin resistance, and the production of free fatty acids which result in increased hydrogen peroxide production [22]. The antioxidative defense mechanism sounds changed throughout pregnancy [6, 23]. Evaluation of the level of different oxidative stress biomarkers provides guidelines for the detection and treatment of pregnancy complications such as miscarriages and PE [22]. We observed that administration of melatonin among pregnant women receiving prophylaxis enoxaparin and aspirin resulted in a significant decrease in NO and hs-CRP, as well as an increase in the serum TAC levels. We did not find any significant effect of melatonin consumption on MDA and PCO levels.

Our investigation revealed that taking melatonin in high-risk pregnant women led to a significant increase in plasma TAC and T-SH levels compared to the control group. Total antioxidant capacity may offer more remarkable scientific clues in comparison to the assessment of single biomarkers because of the cumulative effects of the whole antioxidants in plasma. An advanced antioxidant capability in plasma illustrates the improvement of antioxidant status in the body or the presence of a free radical regulatory process [24]. In agreement with our study, Bouroutzika et al. showed that exogenous melatonin increased TAC levels significantly in people under heat stress [25]. Moreover, in a study by *Mistraletti*, melatonin administration resulted in a considerable rise in TAC levels in intensive care unit (ICU) patients compared to the control group [26]. One study proved that melatonin present in beer contributes to the serum total antioxidant, and controlled beer consumption can protect people against oxidative stress [27]. Free radical scavenging activity of melatonin has been shown in several studies [28–30]. In this regard, it has revealed better performance in comparison with intracellular scavengers such as glutathione and vitamin E [31, 32]. In addition to these direct effects of melatonin, there are also indirect antioxidant effects. Thus, melatonin stimulates GPX activity and inhibits NOS [33]. Total thiol (T-SH) plays an important role in the antioxidant defense system against ROS and other free radicals [34]. In line with our study, T-SH levels increased significantly after melatonin administration in hyperglycemia-induced liver damage in rats [35]. Given the increase in TAC and T-SH levels following melatonin consumption, this research confirmed the antioxidant activity of melatonin shown by previous studies.

Following NOS inhibition, melatonin can decrease the production of free radical nitric oxide. NO itself can be toxic; but in addition, it can be broken down into peroxynitrite anions and finally into the highly toxic OH [33]. Pathophysiology of PE as a multifactorial pregnancy disease could result from abnormal placentation and endothelial dysfunction due to reduced bioavailability of NO. Oxidative stress is suggested to play a critical role in the reduced NO bioavailability in PE pathophysiology through some mechanisms such as inhibiting of eNOS and subsequent defect of NO biosynthesis or via the formation of peroxynitrite by the reaction of NO with the radical anion superoxide [36]. In this study, administration of melatonin from week 6 of pregnancy to delivery resulted in a dramatic decline in the serum level of NO compared with baseline and placebo groups. In a study by Qin et al., results indicated that melatonin considerably inhibited the NOS activity and NO production in LPS-induced acute lung injury in mice [37]. NOS is assumed a prooxidative enzyme, and any factor that decreases its activity would be considered an antioxidant. Melatonin inhibits the activity of NOS, in addition to its NO and peroxynitrite scavenging activity [38].

Exposure of lipids to oxidant compounds leads to stimulation of lipid peroxidation which is a biologically important process, producing various end products such as MDA [39]. We observed that melatonin consumption in the intervention group did not affect serum MDA levels. In contrast with the current study, following melatonin administration in patients with type 2 diabetes mellitus, the serum level of MDA reduced significantly [40]. The same result was obtained in terms of the PCO content. However, in the control group, its level showed a significant decrease at the end of the study compared with the basic feature.

Several studies indicate that inflammation factors and oxidative stress have negative effects on pregnancy and fetal growth. It can lead to the cause of improper implantation of embryos, premature births, low birth weight, birth defects, and miscarriages [11]. We found that using melatonin reduced serum hs-CRP levels throughout pregnancy. In the present work, Jamilian et al. [41] found that 5 mg melatonin administration twice a day for 12 weeks caused a significant reduction in the level of CRP in women with polycystic ovary syndrome. Increased ROS generation can lead to inflammation. Melatonin as an antioxidant can play a pivotal role in preventing inflammatory processes through its free radical scavenging activity [28]. Among the limitations of this study is the small sample size of patients, which suggests that future studies should be conducted with a larger sample size and in different ethnicities.

## 5. Conclusion

Melatonin supplementation from the sixth week of pregnancy until delivery in high-risk women increased the serum levels of TAC and T-SH, as well as reduced NO and hs-CRP but did not influence MDA and PCO levels. This suggests that melatonin administration can improve antioxidant activity as well as reduce inflammation and may provide a useful therapeutic approach for managing pregnancy outcomes associated with ROS production. More investigations with more participants are required to confirm this approach. In addition, further studies should evaluate other biomarkers related to oxidative stress and inflammation to investigate the underlying mechanism of the melatonin effect in ameliorating pregnancy complications caused by oxidative stress more effectively.

## Figures and Tables

**Table 1:** Comparison of changes in the oxidative stress and inflammatory biomarkers for study between the intervention and control groups.

Variable	Groups	Baseline	After	*P* value	Difference (baseline-after)	*P* value
TAC (*μ*mol/L)	Control	663.25 ± 230.36	552.16 ± 135.20	0.036	4.25 ± 553.81	0.008^*∗*^
	Intervention	527.75 ± 142.79	643.34 ± 190.49	0.064	−170.12 ± 322.01	

MDA (*μ*mol/L)	Control	1161.86 ± 195.19	1020.83 ± 130.33	0.043	141.02 ± 290.22	0.396
	Intervention	1179.17 ± 248.04	1125.32 ± 240.10	0.498	53.85 ± 348.80	

T-SH (*μ*mol/L)	Control	9.96 ± 3.03	6.18 ± 1.67	<0.001	3.78 ± 2.67	0.017^*∗*^
	Intervention	10.55 ± 4.02	18.27 ± 30.00	0.658	−7.71 ± 29.39	

NO (*μ*mol/L)	Control	3.13 ± 2.62	2.76 ± 2.70	0.668	0.37 ± 3.78	0.034^*∗*^
	Intervention	4.38 ± 3.84	1.16 ± 1.39	0.004	3.22 ± 4.30	

PCO (*μ*mol/L)	Control	21.10 ± 6.07	6.55 ± 6.66	<0.001	14.55 ± 7.07	<0.001^*∗*^
	Intervention	13.99 ± 6.41	11.51 ± 5.15	0.221	2.47 ± 8.51	

hs-CRP (*μ*g/mL)	Control	12.47 ± 9.82	6.40 ± 3.70	0.067	6.07 ± 11.85	0.278
	Intervention	12.85 ± 9.67	4.38 ± 0.87	0.002	8.46 ± 9.46	

TAC, total antioxidant capacity; MDA, malondialdehyde; T-SH, sulfhydryl protein; NO, nitric oxide; PCO, protein carbonyl; hs-CRP, high-sensitivity C-reactive protein.

## Data Availability

The data supporting the findings of this study are available within the article.
